# Akathisia after chronic usage of synthetic cathinones: A case study

**DOI:** 10.3389/fpsyt.2022.1046486

**Published:** 2022-12-23

**Authors:** Niels Albert, Kirsten Catthoor, Manuel Morrens

**Affiliations:** ^1^Hospital Network Antwerp (ZNA), Antwerp, Belgium; ^2^Faculty of Medicine and Health Sciences, University of Antwerp, Antwerp, Belgium; ^3^Faculty of Medicine and Health Sciences, Collaborative Antwerp Psychiatric Research Institute (CAPRI), University of Antwerp, Antwerp, Belgium; ^4^Scientific Initiative Neuropsychiatric and Psychopharmacological Studies (SINAPS), University Psychiatric Centre (UPC) Duffel, Duffel, Belgium

**Keywords:** synthetic cathinones, akathisia, extrapyramidal symptoms, new psychoactive substances, Barnes Akathisia Rating Scale (BARS), toxicology

## Abstract

**Introduction:**

Synthetic cathinones are a heterogenous group of new psychoactive substances (NPS) with a surging prevalence of use. They are sold under the name of “Bath Salts,” “Legal Highs” or “Research Chemical.” It is a heterogeneous group of substances that have a varying binding selectivity and affinity. Due to limited availability of NPS screening tests, inadequate legislation, the exponential increases in availability of new NPS and the comorbid use of other illicit substances, scientific research remains scarce. As a result, insight in their mental and psychomotor effects is limited.

**Case description:**

We present a case of a 21-year-old woman with daily usage of synthetic cathinones, more specifically α-Pyrrolidinopentiophenone (α-PVP-better known as “Flakka”), α-Pyrrolidinohexiophenone (α-PHP) and alpha-Pyrrolidinoisohexaphenone (α-PHiP). Besides a severe paranoid psychotic state of mind, characterized by persecutory and somatic delusions, there was also a very pronounced psychomotor restlessness during the whole period of hospitalization which was diagnosed as akathisia. She reported that she was unable to sit during meals, had difficulty standing still and felt a constant urge to pay attention to the restlessness. The patient did not take any antipsychotic medication at admission.

**Results:**

The patient was treated in accordance with the current guidelines concerning akathisia with a combination of Quetiapine 200 mg, Propranolol 80 mg, Diazepam 10 mg, Biperiden 4 mg, and Mirtazapine 15 mg without any sufficient alleviation of complaints. Before the start of the treatment, Barnes Akathisia Rating Scale (BARS) score was 11 out of 14 which evolved toward a score of 7 over the course of the 40 day hospitalization, implying persisting severe akathisia which only improved modestly.

**Conclusion:**

The current case suggests that besides cocaine, amphetamines and methamphetamines, synthetic cathinones can also increase the risk for development of extrapyramidal symptoms such as akathisia. Especially a-PVP-analogs as used by the current patient and Pyrovalerone-analogs such as Methylenedioxypyrovalerone (MDPV) are very powerful dopamine reuptake-inhibitors which might lead to strong locomotor activation. Up to this day it remains very difficult to establish a guideline concerning the treatment of intoxication with synthetic cathinones or dependence thereof.

## Introduction

Synthetic cathinones are a group of new psychoactive substances (NPS) with an increasing prevalence of use. They are sold under the name of “Bath Salts,” “Legal Highs” or “Research Chemical.” A cathinone is an alkaloid structure (C9H11NO) and a β-keto analog of amphetamine and thus shares its stimulant effects (1). It is a heterogeneous group of substances that have a varying binding selectivity and affinity (2). Synthetic cathinones act as monoamine reuptake inhibitor through the blocking of the serotonin transporter (SERT), dopamine transporter (DAT) and the norepinephrine transporter (NET). Some synthethic cathinones act as a hybrid compound both blocking the reuptake inhibitor and acting as a substrate of the monoamine receptors thus increasing the mono-amine concentration in the synaptic cleft leading to hyperexcitation of postsynaptic receptors (1).

In addition to sympathomimetic side effects, cardiovascular side effects are also frequently reported (3). Furthermore agitation, anxiety, paranoid delusions, and uncontrollable psychomotor agitation are known side effects (4). Due to limited availability of NPS screening tests, insufficient legislation, the exponential increases in availability of new NPS and the comorbid use of other illicit substances scientific research remains scarce (2). As a result, insight in their mental and psychomotor effects is limited.

Akathisia is a movement disorder characterized by the subjective feeling of inner restlessness or nervousness with a strong urge to move. Examples of such repetitive movements include crossing the legs, rocking or shuffling from one foot to the other (5, 6). The pathophysiology of akathisia has not yet been fully identified, but the basal ganglia and more specifically the Nucleus Accumbens are most often indicated in the development of extrapyramidal movement disorders including akathisia (7).

Clinically, the most used questionnaire to objectify akathisia is the Barnes Akathisia Rating Scale (BARS). This scale consists of four sections that seek to evaluate an objective observation of akathisia, the patient’s awareness of His/Her akathisia, the subjective agitation associated with it and a global clinical assessment of akathisia (8). There is no widely used cut-off for the total score of the BARS, but a global clinical assessment of 2 out of 5 is considered as positive for having akathisia (9).

## Case description

Patient A, a 21-year-old lady with a severe substance use disorder for various substances in addition to a psychotic vulnerability, was involuntarily admitted for a fixed period of 40 days following a paranoid psychotic outburst while being intoxicated. At the time of admission, the patient looked very unkempt. The patient was poorly oriented in time and place, was for the most part uncooperative and had difficulties having a coherent conversation. Sustained attention was affected, and there was logorrhea. There were persecutory and somatic delusions (e.g., She felt as if her eyeballs where placed there by someone else). There were no visual or auditory hallucinations. Furthermore, the patient experienced severe psychomotor restlessness. She reported that she was unable to sit during meals and was continuously rocking back and forth. Finally, there was subconjunctival hemorrhage of unknown etiology.

In addition to the severe psychotic state, the patient found it impossible to eat in a sitting position, found it extremely difficult to stand still and had the feeling that she had to pay attention almost continuously. A Barnes Akathisia Rating Scale (BARS) was taken at the start of treatment, on which the patient scored 11 out of 14 in total and 3 out of 5 on the global clinical assessment, a subscale of the BARS. It was unclear how long the akathisia had already been present. There were no signs of other extrapyramidal symptoms (acute dystonia, parkinsonism or tardive dyskinesia) present. The patient did not take any antipsychotic medication in the 6 months prior to admission. There was no prior history of traumatic brain injury, no neurological antecedents in the patient or (for so far known) her family. Possible differential diagnoses considered for akathisia were ADHD, agitated depression, agitation due to manic or psychotic states and Restless Legs Syndrome but we had insufficient clinical arguments, based on thorough anamnesis and clinical psychiatric examination to withhold any of these diagnoses. There was no history of major mood disorders, nor did she take any antidepressants or mood stabilizers. There was no history of substantial attention deficits or hyperactivity in early childhood and no increase of restlessness in bed or during the night. The restlessness also stayed long after psychotic features disappeared. The patient had no history of psychotic features in absence of substance abuse. As a result, no further screening instruments were used.

Anamnestically we learned that from the age of 17, there was also heavy Ketamine use (up to 8 grams per day) for which there were several episodes of emergency presentation with abdominal cramping, falling within the phenomenon of k-cramps: Biliary obstruction and dilatation in patients with heavy ketamine usage. In the past there was also sporadic use of cocaine, amphetamines, 3,4-methylenedioxy-methamphetamine (MDMA), 4-Bromo-2,5- dimethoxyphenethylamine (2-CB), gamma hydroxybutyrate (GHB) and heroin. Earlier emergency reports do not mention similar akathisia symptoms, nor did the patient mention experiencing them during the usage of these substances. She stopped taking before mentioned substances around 3 years prior to current hospitalization, when she switched to synthetic cathinones. Previous psychiatric assistance was limited to a 21-day crisis admission, 6 months before the current admission because of a substance induced paranoid psychotic episode, in the absence of akathisia or any other psychomotor symptoms. Psychotic symptoms were treated with aripiprazole 30 mg, leading to an alleviation of the symptoms, but the treatment was immediately discontinued after hospitalization.

There was also a significant familial burden: The father had been alcohol abstinent for about 2 years and had also been dependent on amphetamines and cocaine in his youth. Mother had known for 25 years varying periods of heroin addiction and methadone dependence. When she was born, the patient was thus hospitalized for an extended period because of methadone withdrawal.

Routinely toxicology testing for stimulants such as cocaine, amphetamine and methamphetamine but also cannabis, benzodiazepines and opiates repeatedly showed negative during current hospitalization, but the patient admitted to having used a combination of several synthetic cathinones before admission, and she managed to use these substances once during hospitalization. For about 3 years she smoked mainly α-Pyrrolidinopentiophenone (α-PVP—better known as “Flakka”), α- Pyrrolidinohexiophenone (α-PHP) and alpha-Pyrrolidinoisohexaphenone (α-PHiP) on daily basis with peaks up to 6 to 7 grams per day. There was also occasional use of other synthetic cathinones being Mephedrone (“Miaow Miaow” or “MCAT”), Hexedrone, 4-Chloro-alpha-pyrrolidinovalerophenone (4-Chloro-PVP) and 4’-Fluoro-α-pyrrolidinopentiophenone (4-Fluoro-α-PVP).

**FIGURE 1:**
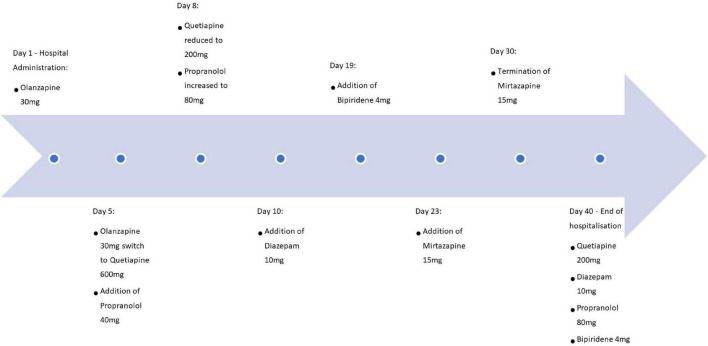
Timeline of pharmacological treatments.

Figure 1 represents a summarizing timeline of pharmacological treatments. The psychotic features were initially treated for 5 days with Olanzapine up to 30 mg daily but given persistent complaints of motor restlessness was switched initially to Quetiapine 600 mg and was diminished to 200 mg daily. Quetiapine was administered for 35 days (the remainder of the hospitalization period) under which the psychotic features stabilized. Propranolol was also associated for the whole period of hospitalization (40 days) and was increased to 80 mg while monitoring her vital parameters. Due to limited effects on akathisia, additionally Diazepam 10 mg was given at day 10 and was continued for 30 days. Despite these interventions, akathisia remained present, resulting in the addition of Biperidene 4 mg at day 19, which was continued during the last 21 days of hospitalization. Mirtazapine 15 mg was briefly associated without convincing effect. Intake of the medication was systematically supervised by nursing staff. The BARS score stayed high at discharge, with a total score slightly dropping to 7 in total and 2 out of 5 at the global clinical assessment subscale. The patient left the hospital at her own request at the end of her involuntary admission, despite the presence of persisting symptoms and as a result, some treatment options were left unexplored.

Despite the administration of propranolol, a pulse of 100–130 beats per minute was consistently measured, followed by an electrocardiographic check-up. The electrocardiogram was reassuring and showed a sinusoidal rhythm with non-specific T-top inversions in the inferior leads II, III, and aVF. To exclude epileptogenic activity and neurotoxicity, a control EEG and brain MRI were also performed: Both could not retain any abnormalities. The biochemical workup and blood pressure also showed reassurance. There was no sign of inflammation (CRP 1.8 mg/L and WBC 7.16 × 10*3/μg), no deviations in electrolytes (Sodium 140 mmol/L, Potassium 4.1 mmol/L, Chloride 102 mmol/L and Bicarbonate 25.6 mmol/L) and no thyroid abnormalities (TSH 0.548 mIU/L, Free T4 12.9 pmol/L). The differential diagnoses of hyperactive delirium and neuroinflammation were therefore excluded.

## Synthetic cathinones

Cathinone is the psychoactive substance found in the plant Catha Edulis (Khat), which grows primarily in the horn of Africa and the Arabian Peninsula (10). The leaves of the plant are chewed, releasing the psychoactive substance. A cathinone is an alkaloid structure (C9H11NO) and a β-keto analog of amphetamine and thus shares its stimulant effects (1). The first synthesized cathinones were created in the 1920 s because of their potential as antidepressants and appetite suppressants. For example, Methcathinone became the first commercialized synthetic cathinone to be marketed as an antidepressant in 1930 and 1940 s primarily in the Soviet Union (2). Afterward, Mephedrone, Pyrovalerone, Amfepramone, and Methylone were also investigated as agents for chronic fatigue, excessive appetite and Parkinson’s disease respectively. Currently, Bupropion is the only synthetic cathinone still commercially available.

Methylone which was never commercialized—unlike Pyrovalerone and Amfepramone—was the first synthetic cathinone to be traded on European black markets as a recreational stimulant at the turn of the century. This was because of its potential psychostimulant effect similar to MDMA. Shortly thereafter 3-Fluoromethcathinone (3-FMC), 4-Fluoromethcathinone (4-FMC), Butylone and Methylenedioxypyrovalerone (MDPV) also appeared on the circuit. They produce a euphoric feeling with increased empathy, openness, social reciprocity and libido. Their popularity has also been sustained because of high cocaine prices as well as reduced purity of amphetamines and the lack of proper screening to confirm use (2, 11). They are sold under the common name “Bath salts,” “Research chemicals” or “Legal highs” (1).

The latter name was given to them mainly because of the lagging legislation. In 2010 mephedrone was the first synthetic cathinone to be listed in legislation as an illegal drug. In the following years, however, more new cathinones were developed than there were added to the legislation. In 2011, 8 new agents were released, while 69 similar derivatives found their way to recreational users between 2012 and 2015 (2).

There is an increasing prevalence of use of NPS. Recent data by the European Monitoring Center on Drugs and Drug Addiction (EMCDDA) suggests that up to 5% of tested youth have experienced with these new substances (12). NPS’s are especially popular among “clubgoers” and “psychonauts” with a pooled prevalence of 20% in certain populations (13). The world wide web serves as a primary source of information about these drugs. Users can obtain information about new products through online forums, chat rooms and blogs but also buy them in online shops where consumers are targeted by aggressive marketing strategies (attractive names, colorful packaging and free samples). Multiple of these websites also contain medical misinformation making them a possible public health risk. Cyberspace could however also be an excellent environment for information campaigns aiming at prevention of NPS abuse (14, 15).

Synthethic cathinones can be ingested in a variety of ways, but the most common distribution forms are: oral (tablet, pill, liquid), nasal, intravenous as well as inhalation as a gas. The half-life of synthetic cathinones averages from 1.5 to 4.3 h. In comparison, the half-life of cocaine is about 1 h while that of amphetamine is 10–13 h (16). Cathinones are for the most part excreted through the urine and despite its strong psychoactive effect it has only limited blood-brain barrier permeability, presumably due to the low lipophilic nature of the substance (2). This makes users more easily inclined to overuse or use higher dosages compared to amphetamine.

Synthetic cathinones act as monoamine reuptake inhibitor through the blocking of the SERT, DAT and the NET thus increasing the mono-amine concentration in the synaptic cleft. Some cathinones also directly release Serotonin, Dopamine or Noradrenaline (1). Although they have a similar chemical structure, their binding strength, selectivity and affinity are very different from each other. They are also subdivided according to this selectivity and affinity. A first group consists of substances with selective SERT inhibition (such as MDMA) or non-selective SERT, DAT or NET inhibitors (such as cocaine). Examples include 4-Methylethcathinone (4-MEC), mephedrone, ethylone, butylone, pentylone, methylone, 3,4-Dimethylmethcathinone (3,4-DMMC), and methedrone. A second group consists of substances with selective inhibition for DAT, with also potent inhibition of NET (such as methamphetamine). Among others, buphedrone, fephedrone, methcathinone, and pentedrone belong to this category. A third group are the synthetic cathinones that are considered extremely potent inhibitors of DAT and NET, with negligible binding to SERT. Pyrovalerone, α-PVP, MDPV, 3′,4′-Methylenedioxy-α-pyrrolidinobutyrophenone (MDPBP) and 3′,4′-Methylenedioxy-α-pyrrolidinopropiophenone (MDPPP) belong to this category (2). Consequently, their high DAT/SERT ratio makes the latter category the most addictive (and toxic) (17).

In addition to sympathomimetic side effects (blurred vision, dry mouth, hyperthermia and mydriasis), cardiovascular side effects are also frequent (increased blood pressure, tachycardia, arrhythmias and coronary spasms) (3). Agitation, anxiety, paranoid delusions, and uncontrollable psychomotor agitation are also known side effects (4).

## Akathisia

Akathisia is a movement disorder characterized by the subjective feeling of inner restlessness or nervousness with a strong urge to move. Examples of such repetitive movements include crossing the legs, rocking or shuffling from one foot to the other (5, 6). The phenomenon was described in 1901—before the existence of antipsychotics—as the inability to sit down, but then ended up under the category of extrapyramidal symptoms (18).

Akathisia is the most common and disabling movement disorder that can occur as a side effect of antipsychotics. Studies indicate a prevalence of 15–35% in patients with schizophrenia, with the risk being higher for patients treated with a first generation antipsychotic (FGA) than those treated with a second generation antipsychotic (SGA) (18). Patients treated with a combination of SGAs had a higher risk than those treated with a combination of an FGA and an SGA (34.2 vs. 14.7%) (18). In addition, patients treated with more than one SGA had a risk up to three times greater than patients treated with monotherapy SGA (34.2 vs. 10.9%) (9). Other products that may promote akathisia include Selective Serotonin Reuptake Inhibitors (SSRIs), Monoamine Oxidase Inhibitors (MAO-I’s), Tricyclic Antidepressants (TCAs), antibiotics, calcium channel blockers, and drugs such as cocaine, amphetamine, and methamphetamine (10, 19–21). Finally it can also be caused by Parkinson’s disease, post-encephalitis parkinsonism or traumatic brain injuries (22, 23).

The pathophysiology of akathisia has not yet been fully identified, but the basal ganglia and more specifically the Nucleus Accumbens are most often indicated in the development of extrapyramidal movement disorders including akathisia. The classic hypothesis is an imbalance between the dopamine and serotonin, noradrenaline, acetylcholine and GABA neurotransmitters (7). There is an inhibition of dopaminergic neurotransmission (regulated by other neurotransmitters such as serotonin and GABA) due to receptor blockage which is the case in antipsychotic induced akathisia where up to 80% of D2 receptors are blocked (7). Other possible mechanisms are dopamine depletion due to substance abuse, but there is also evidence of over stimulation of the locus coeruleus, neuroinflammation, or damage to the blood-brain barrier (24).

## Discussion

In our case study, we encountered a 21-year-old lady with a substantial dependence on synthetic cathinones, pronounced psychotic symptomatology and severe, difficult-to-treat akathisia. The treatment of akathisia broadly consists of two possible interventions: Modifying (or reducing) the antipsychotic used and adding an agent specifically against akathisia. Suggested antipsychotics that would reduce akathisia are Quetiapine and Chlorpromazine or in second line Clozapine. Suggested agents specifically against akathisia are Propranolol (40–80 mg), Mirtazapine (15 mg) or Biperidene (2–6 mg). In second line, Amantadine (100 mg) or Clonidine (up to 0.15 mg), Lorazepam (1–2 mg), Clonazepam (0.5–1 mg) or Diazepam (5–15 mg) or Mianserine (15 mg) (5, 18). Pregabalin, Gabapentin, Vitamin B6 and N-acetylcysteine also showed some effect in some studies (25–27).

For the current patient, it was appropriate to reduce the dose of antipsychotics as well as opt for a relatively low-risk SGA (Quetiapine) for the treatment of the remaining psychotic features. The delusions diminished considerable and were less overt but where never fully absent. Clozapine was not considered since the psychotic symptoms were significantly influenced by substance abuse. In addition, our patient was treated with Propranolol 80 mg, Biperidene 4 mg and Diazepam 10 mg resulting in some improvement but no complete absence of akathisia. Since the patient was unable to remain abstinent for at least a month—she fled the department once after 18 days—the diagnosis of tardive dyskinesia or tardive akathisia after abuse of synthethic cathinones could not be made despite of correct treatment of the akathisia. Although the BARS scored positive for akathisia, a phenotype of excited catatonia secondary to usage or withdrawal of synthetic cathinones could unfortunately not be excluded (28). In that case, subsequent treatment would consist of high doses of benzodiazepines (without simultaneous administration of other pharmaceuticals) and perhaps even electroconvulsive therapy (ECT) (29).

Synthetic cathinones are a heterogeneous and diverse group of substances, each with a distinct mode of action and receptor binding affinity. Some cathinones are pure uptake inhibitors of dopamine, serotonin or norepinephrine, while others also release dopamine, serotonin or norepinephrine. Especially the pyrovalerone analogs including MDPV with a DAT/SERT inhibition ratio of more than 100 and α-PVP analogs and have a very powerful dopamine reuptake inhibition which can lead to strong locomotor activation (1). Like cocaine, amphetamine and methamphetamine, synthetic cathinones also seem to be able to give rise over time to various movement disorders such as tremor, gait disorders, parkinsonism and hyperkinetic disorders such as dyskinesias, dystonias and akathisia. However, research in this area is mainly limited to animal studies, *in vitro* studies and case reports, partly due to the limited possibility of testing, rapid increase of new substances and often comorbid use of other drugs.

## Patient perspective

The patient fully supported the suggested evidence-based treatment options. She did however not wish to stay longer in the hospital than legally imposed. The patient was supportive for the publication of her case and signed an informed consent.

## Conclusion

Synthetic cathinones are a broad group of products. They all have stimulatory effects, but nevertheless vary greatly in their binding strength, selectivity and affinity. Rapid development of new products, limited testing opportunities and lagging legislation make them difficult to detect, investigate and regulate. To varying degrees, they may pose a risk of developing extrapyramidal symptoms such as akathisia. An early and correct interpretation of akathisia is important given that a misinterpretation of akathisia as general agitation can lead to increasing the dosage of antipsychotics with a paradoxical worsening of symptomatology.

## Data availability statement

The original contributions presented in this study are included in the article/supplementary material, further inquiries can be directed to the corresponding author/s.

## Ethics statement

Ethical review and approval was not required for the study on human participants in accordance with the local legislation and institutional requirements. The patients/participants provided their written informed consent to participate in this study. Written informed consent was obtained from the individual (s) for the publication of any potentially identifiable images or data included in this article.

## Author contributions

NA treated the patient, wrote the case report, and collaborated in literature research. KC was the leading treating clinician, collaborated in literature search, and reviewed the case report. MM collaborated in literature research, and reviewed the case report. All authors contributed to the article and approved the submitted version.
